# Testing the efficacy of a brief sexual risk reduction intervention among high-risk American Indian adults: study protocol for a randomized controlled trial

**DOI:** 10.1186/s12889-016-3040-y

**Published:** 2016-04-29

**Authors:** Rachel Chambers, Lauren Tingey, Anna Beach, Allison Barlow, Anne Rompalo

**Affiliations:** Johns Hopkins Center for American Indian Health, Johns Hopkins University, 415 North Washington Street Suite 400, Baltimore, Maryland 21224 USA; Johns Hopkins School of Medicine, Johns Hopkins University, 415 North Washington Street Suite 400, Baltimore, Maryland 21224 USA

**Keywords:** Sexually transmitted infection, American Indian, Risk-reduction, Counseling, Substance use, Suicide ideation, Prevention

## Abstract

**Background:**

American Indian adults are more likely to experience co-occurring mental health and substance use disorders than adults of other racial/ethnic groups and are disproportionately burdened by the most common sexually transmitted infections, namely chlamydia and gonorrhea. Several behavioral interventions are proven efficacious in lowering risk for sexually transmitted infection in various populations and, if adapted to address barriers experienced by American Indian adults who suffer from mental health and substance use problems, may be useful for dissemination in American Indian communities. The proposed study aims to examine the efficacy of an adapted evidence-based intervention to increase condom use and decrease sexual risk-taking and substance use among American Indian adults living in a reservation-based community in the Southwestern United States.

**Methods/Design:**

The proposed study is a randomized controlled trial to test the efficacy of an adapted evidence-based intervention compared to a control condition. Participants will be American Indian adults ages 18–49 years old who had a recent episode of binge substance use and/or suicide ideation. Participants will be randomized to the intervention, a two-session risk-reduction counseling intervention or the control condition, optimized standard care. All participants will be offered a self-administered sexually transmitted infection test. Participants will complete assessments at baseline, 3 and 6 months follow-up. The primary outcome measure is condom use at last sex.

**Discussion:**

This is one of the first randomized controlled trials to assess the efficacy of an adapted evidence-based intervention for reducing sexual risk behaviors among AI adults with substance use and mental health problems. If proven successful, there will be an efficacious program for reducing risk behaviors among high-risk adults that can be disseminated in American Indian communities as well as other rural and under-resourced health systems.

**Trial Registration:**

Clinical Trials NCT02513225

## Background

Substance use is a major public health problem in the United States. In 2013, 9 % of U.S. adults used illicit drugs and 25 % were binge alcohol users [1]. Comorbid mental health problems are a major contributing factor to substance use; it follows that almost one in four U.S. adults with serious mental health problems also have a substance use problem [2–4].

American Indian (AI) adults shoulder a greater burden of both substance use disorder and poor mental health relative to adults of other races/ethnicities [1, 5, 6]. In 2014 the rate of co-occurring mental health and substance use disorders was 2.6 times higher among American Indian/Alaska Native (AI/AN) adults than the national average [6]. Research conducted with individual AI communities corroborate national data and illustrate correlating risks for substance use, depression and suicide [7–9].

Substance use and poor mental health are linked to greater risk-taking and sensation-seeking, in particular sexual risk behaviors [8, 10–12]. Although there is a dearth of research examining sexual behavior among AI adults, high-risk behaviors including inconsistent condom use, early age of sexual initiation and multiple partners have been reported among AI adolescents [13].

In 2010, national AI/AN Gonorrhea and Chlamydia rates were 4.6 and 4 times the rates among Whites [10]. In 2013 in Arizona, where this study will take place, AIs had the highest rate of chlamydia of all races/ethnicities. Between 2012 and 2013 the gonorrhea rate among AIs in Arizona increased by 23 %, and accounted for 9.7 % of all reported infections [14]. Data collected from a previous study conducted by the research team with 322 AI adolescent mothers living in reservation-based communities in Arizona highlight the intersection of substance use, mental health and sexual risk: female participants reporting a lifetime sexually transmitted infection (STI) had significantly higher depressive symptoms than women without (CESD score of 15.5 vs. 13.2, *p* = 0.05). Further, women in this sample with a recent episode of drunkenness were more likely to have a lifetime STI diagnosis (39 % vs. 23 %, *p* = 0.04) as were those with recent marijuana use (45 % vs. 22 %, *p* < 0.01).

Research conducted by the Indian Health Service (IHS) suggests higher STI rates in many AI/AN communities are partly attributable to profound healthcare access barriers [15–17]. Nearly 40 % of AI/AN adults rely solely on IHS, the entity responsible for providing and funding health care services in AI/AN communities. Historically underfunded, IHS has limited capacity to provide healthcare services, particularly substance use and mental health treatment [18, 19]. Much of this is attributed to a shortage of healthcare providers; in the IHS system there are approximately 4 psychologists per 100,000 patients, one sixth of the number of psychologists available to the general U.S. population [20].

In AI/AN communities that are rural and/or reservation-based, difficulty obtaining care is worsened by reduced ability to access IHS clinics. In a 2005 report, 8 out of 13 IHS facilities had patients traveling 60+ miles one-way for treatment [21]. With high rates of AI/ANs living in poverty, many may lack reliable transportation to travel long distances to IHS. Not surprisingly, AIs are two times more likely than Whites to report transportation barriers to healthcare (39 % vs. 18 %) [22]. Additional obstacles, especially in small close-knit communities include confidentiality concerns and stigma. Patients may be uncomfortable seeking care at IHS facilities due to friends or relatives employed there, especially for STIs and/or HIV which are still highly stigmatized in many AI communities [10, 23, 24]. Taken together, identified access barriers lead to reduced STI screening and disease identification, missed appointments and treatment noncompliance, as well as delayed care [22, 25, 26].

### The current study

Developing and evaluating interventions to address the confluence of poor mental health, substance use and risky sexual behaviors while simultaneously reducing healthcare access barriers in AI communities is a laudable public health goal [17]. Yet, scant programming exists targeting these interrelated risks, particularly for AI adults residing in rural reservation-based settings where STI screening rates are low and prevalence high [27, 28]. This study will assess the efficacy of an adapted, evidence-based intervention, called EMPWR, for decreasing substance use and risk for STIs, and improving mental health and access to STI screening. This paper describes the process of intervention adaptation and protocol for EMPWR’s implementation and evaluation.

#### EMPWR program

Recognizing the balance between developing interventions from the ground up versus adapting existing evidence-based interventions (EBI), it was determined that an evidence-based program would be selected for adaptation [29–32]. Various evidence-based programs were reviewed by the research team and members of the participating AI community for previous evidence base and potential feasibility. This review in conjunction with consultation with local stakeholders and assessment of community readiness to address sexual risk as well as STI screening led to the selection of Project RESPECT for implementation. Project RESPECT is an EBI targeting HIV testing uptake and designed to support client-centered risk reduction. It uses “teachable moments” to motivate individuals to explore recent risk behaviors and enhance their perceptions of self-risk. Project RESPECT was evaluated with STI clinic patients through a multisite efficacy trial. Results from this trial indicated Project RESPECT participants had lower STI incidence and higher self-reported condom use than controls [33].

To adapt Project RESPECT, the study team sought guidance and input through seven gender specific focus group discussions (male *n* = 3) with at-risk AI adults from the participating community and three community advisory board meetings comprised of key stakeholders and tribal leaders. The adapted intervention was renamed by tribal partners: Educate, Motivate, Protect, Wellness, and Respect (EMPWR).

Four main adaptations were made to Project RESPECT: 1) The intervention setting was changed from a clinic to a home or community-based setting. This adaptation eliminates the need for participants to travel to an IHS clinic and reduces potential for confidentiality concerns. 2) Trained paraprofessional community health workers and not clinic-based STI counselors will deliver the intervention. With high unemployment rates, many AI communities have a ready workforce of highly-skilled paraprofessionals who can fill gaps in healthcare access and service delivery. Further, paraprofessionals from the participating community are culturally matched and socio-contextually suited to deliver the EMPWR curriculum. 3) Self-administered sample collection for STI screening, as opposed to clinic-based HIV testing will be offered. Self-administered STI screening has been implemented in various communities in the U.S. and previously piloted with young AI adults from the participating community. Pilot results indicated this method was well-received; approximately 80 % of participants with previous STI testing experience preferred this method over visiting a hospital or clinic [34]. Also, community feedback indicated widespread HIV stigma. Recognizing the importance of community readiness, the intervention-related screening option was changed from HIV to treatable STIs (*Neisseria gonorrhea*, *Chlamydia trachomatis* and *Trichomonas vaginalis*). 4) The target population for the intervention was changed from STI clinic patients to AI adults with identified substance use and/or mental health problems for study participation. This choice reflects the co-occurring health disparities previously described, and the understanding that those most at-risk are often less motivated to seek formal help [35, 36]. Participants will be recruited from an innovative, tribally-mandated community-based surveillance system that tracks high-risk behaviors [37–39]. Recruitment from this system may be an important strategy for engaging those typically absent in research and outpatient care.

#### Study aims

This study will be conducted with AI adults who are substance users and have co-morbid mental health problems living in a rural, reservation-based community in the Southwestern U.S. The study will be carried out through a two-arm randomized controlled trial design to test the efficacy of the EMPWR program to measure changes in condom use, STI screening uptake, substance use patterns and mental health status. This will enable conclusions to be drawn about the intervention’s impact on individual risk factors, providing insight into the most appropriate application of the intervention. Additionally, by looking at the combination of substance use, mental health and sexual risk taking, the study team will be able to assess how these risk factors interact with one another, and specifically how the implementation of risk reduction strategies is dependent upon mental health and substance use.

Primary research questions include: 1) Is the intervention effective in increasing condom use at last sex; 2) Is the intervention effective in increasing uptake of STI screening; 3) Is the intervention effective in decreasing substance use; and 4) Is the intervention effective in improving mental health status? Secondary research questions include: 1) How does substance use impact STI screening and treatment seeking behaviors; and 2) How does mental health status impact STI screening and treatment seeking behaviors?

## Methods/Design

### Overview and hypotheses

**Fig. 1:**
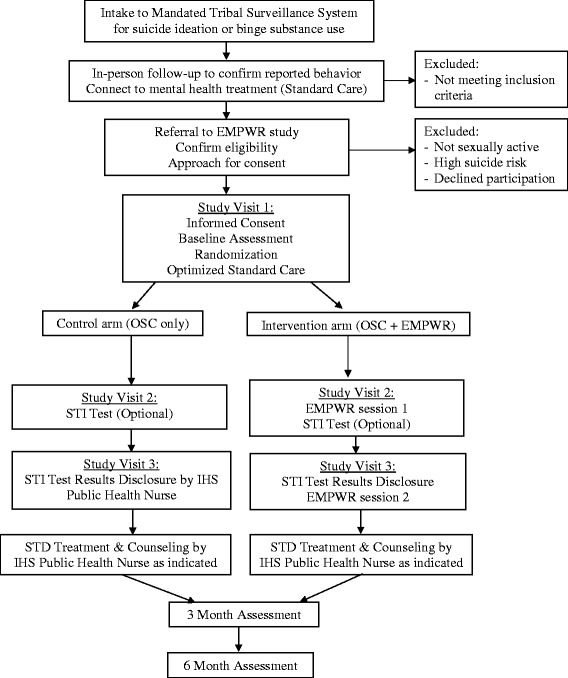
EMPWR Trial Design

A two-arm randomized controlled trial (RCT) will be used to test the efficacy of the EMPWR intervention to increase high-risk adult’s condom use at last sex compared to a control group (see Fig. 1). The intervention consists of two sessions which aim to enhance participant’s sense of self-risk, identify practical risk-reduction steps, and reinforce positive behavior change through a risk-reduction plan. The study team hypothesizes that there will be an increase in condom use following delivery of the two sessions. At 3 months follow-up, the study team hypothesizes that there will be a higher rate of condom use during sex amongst participants in the intervention group compared with the control group. At 6 months follow-up, condom use will again be measured to see if any increases were maintained over time. The study team hypothesizes any effects of the intervention on condom use behavior will be mediated by participant’s substance use and mental health status.

The study will be conducted by the participating tribal community in partnership with Johns Hopkins Center for American Indian Health (JHU). The Tribe resides on a Reservation in rural Northeastern Arizona. The study design was approved by the Tribe’s governing Tribal Council and Health Advisory Board as well as the JHU and IHS research review boards. This manuscript was approved by the Tribal Council and Health Advisory Board.

### Recruitment and consent

Participant inclusion criteria includes: AI ethnicity, 18–49 years of age, member of the participating tribal community, written informed consent, sexually active within the last 3 months, and at least one episode of binge substance use and/or suicidal ideation in the last 90 days. Participants will be recruited through the tribe’s mandated community-based surveillance system which tracks all incidents of binge substance use and suicidal ideation [37, 38]. The surveillance system defines binge use as: that which results in serious consequences to the individual such as losing consciousness and/or requiring medical attention for complications associated with a high blood alcohol level or drug toxicity level. The surveillance system defines suicide ideation as: thoughts to take one’s own life with or without preparatory action. Reports of binge use and suicide ideation are made to the surveillance system using standardized forms. Surveillance staff is authorized by tribal law to conduct in-person follow-up visits with every reported individual to confirm the event, gather more detailed information, and triage the individual to available outpatient care [37, 38]. During this in-person visit, surveillance staff will screen potential participants for study eligibility criteria. Individuals meeting eligibility criteria and who express interest in study participation will be referred to a study team member to complete informed consent and enrollment.

### Randomization and sample size

This study will use a randomized controlled group design; the unit of randomization will be the individual. Unique participant identification numbers will be used to randomize individuals to one of two study groups: intervention or control. A 1:1 allocation ratio and stratified randomization technique will be used to ensure a 1:1 allocation of study conditions across males and females and age group (i.e., 18–29 and 30–49). Study group allocation will be revealed to participants after completing the baseline assessment.

Sample size and statistical power estimates are predicated on the primary hypothesis that our intervention will increase self-reported condom use in the intervention group compared to the control group. A sample size of 240 (120 per study group) will provide >80 % power to detect a 20 % difference in condom use at last sex where alpha = 0.05. The effect size is based on observed impacts in past trials of the original EBI (Project RESPECT) [33, 40]. The study team will over-recruit to allow for participant withdrawal and drop-out between the baseline and 6-month follow-up. Based on retention rates from past randomized controlled trials conducted by the study team with the target population, it is estimated that 300 participants will need to be recruited to retain a final sample size of *n* = 240 [34, 41, 42].

### Intervention

EMPWR is a client-focused counseling intervention based on the Theory of Reasoned Action, Social Cognitive Theory, and the Indigenist Stress-Coping Model [33, 40, 43, 44]. EMPWR aims to assess participant’s risks, enhance participant’s perception of self-risk, identify barriers to risk reduction, and negotiate a tangible risk-reduction plan. EMPWR consists of two, ~40 minute lessons delivered one-on-one approximately 2 weeks apart by a trained paraprofessional interventionist from the participating tribal community.

In the first session, study staff help participants understand their personal risk factors for STIs (i.e., substance use, mental and emotional health, sexual health, etc.) and develop achievable personalized risk-reduction steps that emphasize individual and community level strengths and resources. STI screening through self-administered urine sample collection (for *Neisseria gonorrhea*, *Chlamydia trachomatis* and *Trichomonas vaginalis*) is offered at the end of the first session.

In the second session, study staff will discuss the STI test result and provide additional counseling to support client-initiated behavior change since the first session and longer-term risk-reduction planning. All participants who test positive for a STI will receive an assisted referral to a Public Health Nurse at the local IHS hospital.

Fidelity to the EMPWR curriculum will be assessed through a monitoring checklist completed by senior study team members post-intervention delivery upon reviewing audio and video files of curriculum implementation. Feedback will be provided to study staff according to fidelity monitoring checklist criteria; additional curriculum training will be delivered as necessary.

### Control condition

The control condition will consist of Optimized Standard Care (OSC) alone. Standard Care consists of the referral to outpatient care (mental health and/or substance abuse treatment) received by all individuals reported to the tribal surveillance system. OSC will include educational pamphlets and provision of information on substance use, signs and symptoms of mental health problems, and information about STI screening resources. STI screening through self-administered urine sample collection and an assisted referral to treatment for those testing positive will also be offered to the control group. OSC will be delivered to both intervention and control groups so that any between-group differences can be attributed to the EMPWR intervention.

### Data collection

Measures will be delivered at baseline, 3 months, and 6 months post-intervention completion. Data will be collected in participant’s homes or another private location via laptop computer utilizing Audio Computer Assisted Self-Interview (ACASI), which has been found to gather more accurate reports of sensitive behaviors among the participating AI community, as compared to traditional interview or self-administered paper questionnaires [45]. Participants will be given $15, $15 and $20 gift cards after completion of the baseline, three and 6 month evaluations, respectively. All measure items were piloted with two males and two females from the participating community to assess comprehension, language, and cultural relevance. Edits were made accordingly.

Selected measures will assess intervention impact on: 1) sexual risk behaviors including condom use at last sex, 2) substance use and associated risks and behaviors, 3) mental health outcomes including anxiety, depression, and suicidal thoughts or behaviors, 4) environmental stressors including historical loss and discrimination, and 5) treatment-seeking behaviors. Please see Table 1 for a description of the measures to be utilized.

**Table 1:** EMPWR Program Evaluation Measures

Measures	Description of measure
EMPWR Questionnaire	Assesses sexual behavior and related risk factors through close-ended questions that asks about STD and sexual behavior history, knowledge and attitudes. Adapted from that which was implemented in the original trial of RESPECT [33].
Historical Loss Scale	12-item assessment that asks questions about losses that may be experienced by AI adults and rate how frequently the loss comes to mind [46].
Care Seeking Scale	4-item scale developed by the research team to assess health-seeking behaviors and preferences for medical care.
Enculturation and Discrimination Scale	27-item scale assessing the extent to which a person is embedded in their culture, self-reported cultural identify and perceived discrimination [47].
Spiritual Coping Scale	1-item that asks the extent to which a participant’s spirituality is involved in their understanding of or dealing with stressful situations.
Suicide and Self-Injury Risk Assessment	Adapted from two suicide assessments, the Columbia Suicide Screen [48] and the Suicide Intent Scale [49], it consists of 17-items evaluating past suicidal and self-injurious behavior.
Brief Symptom Inventory	53-item psychological self-report symptom scale that measures a person’s overall psychological distress level and the number and intensity of symptoms [50].

### Outcomes

#### Primary outcome

The primary outcome for this trial will be condom use at last sex.

#### Secondary outcomes

Secondary outcomes for this trial will include evaluating over the last 3 months: 1) number of sexual partners; 2) use of alcohol, drugs, and/or other substances; 3) binge substance use; 4) suicidal and self-injurious thoughts and behaviors; 5) number of symptoms of mental health problems; 6) initiation of self-administered sample collection for STI screening; 7) follow-up with STI treatment (if testing positive).

### Statistical analysis

Summary scores of outcomes will be stratified by participation in the intervention versus control groups and compared using chi-square tests, for binary or categorical outcomes, and t-tests and analysis of variance (ANOVA) for continuous outcomes. The equivalence of the two study groups at baseline will be compared across a range of background variables. Study hypotheses will be tested using an “intent to treat” model. Participants who drop-out during the study and don’t provide at least 3-month follow-up data will be removed from the analysis. Outcomes will be evaluated using generalized linear mixed models (GLMM), commonly used in longitudinal data analysis for both binary and continuous outcomes. If EMPWR is effective in increasing condom use at last sex, mediation analysis will be conducted to identify whether changes in substance use and mental health symptoms are responsible for this change.

## Discussion

This study protocol presents one of the first randomized controlled trials to assess the efficacy of an adapted evidence-based intervention for reducing sexual risk behaviors among AI adults with substance use and mental health problems. A limitation of the study is that sexual behavior, substance use and mental health are measured by self-report. While self-report is the most widely used methodology to assess behavior change, it is not without biases. This study utilizes a computerized assessment administration (ACASI) which has shown to mitigate these potential biases [45]. Also, we can triangulate self-reported outcomes with STI incidence biomarkers. Another limitation is a short follow-up period; while typical of behavior change research we may have less capability to demonstrate long-term intervention efficacy. Next, the surveillance system from which we will recruit is unique to the participating community and may not be replicable in other contexts. Also some individuals may not associate their mental health or substance use problems with sexual risk-taking and may be less inclined to enroll in the study. Study staff are trained to discuss the relationship between substance use, mental health and sexual risk taking when a person is approached for participation.

There are many strengths of this study including the use of an evidence-based intervention adapted to fit the context of the participating community while keeping the core components intact. If EMPWR proves feasible, acceptable and efficacious, the process utilized to adapt Project RESPECT may be translated to other communities or other EBIs. Another strength of the study is that it aims to engage a high-risk population who may not be captured in the IHS system. Further, the client-centered approach allows tailoring to an individual’s readiness for behavior change. Including self-administered sample collection for STI screening, proven feasible and acceptable in the participating community, further breaks down healthcare access barriers. Finally, the design of EMPWR allows for easy uptake and dissemination by health systems through community health worker programs. This includes IHS’s Community Health Representative and Public Health Nursing departments.

To our knowledge, this is the first randomized controlled trial of a brief risk-reduction intervention paired with self-administered STI screening in an AI community. If EMPWR proves efficacious, there will be a program for AI adults with mental health and substance use problems to reduce their high-risk behaviors. Additionally, we may deepen our understanding of the relationship between sexual behavior, substance use, and mental health risk which may be beneficial to other AI communities and high-risk populations.
